# The effect of psychotherapeutic interventions on pain and quality of life in endometriosis: a systematic review and meta-analysis study

**DOI:** 10.1007/s11845-025-03985-6

**Published:** 2025-06-28

**Authors:** Handan Özcan, Ayşe Çuvadar, Sevda Uzun

**Affiliations:** 1 Hamidiye Faculty of Health Sciences, University of Health Sciences, Istanbul, Turkey; 2 Department of Midwifery, Faculty of Health Sciences, Karabuk University, Karabuk, Turkey; 3 Department of Psychiatric Nursing, Faculty of Health Sciences, Gumushane University, Gumushane, Turkey

**Keywords:** Endometriosis, Pain, Psychotherapeutic intervention, Quality of life

## Abstract

**Aim:**

This study aimed to determine the effect of psychotherapeutic interventions on pain, quality of life and health profile in women diagnosed with endometriosis.

**Methods:**

For this systematic review and meta-analysis study, studies were obtained by searching PubMed, Web of Science, EBSCOhost, Google Scholar, and YÖK Thesis Center databases between August and October 2024 without any year limitation. After the review, 10 studies were included in the study. The data were synthesized by meta-analysis and narrative methods.

**Results:**

In this systematic review and meta-analysis, psychotherapeutic interventions for the treatment and management of endometriosis were found to be effective on quality of life (SMD = 0.515, 95% CI = 0.035 to 0.995; *Z* = 2.104, *I*^2^ = 67.523%, *p* < 0.05), pain (SMD =  − 0.454, 95% CI =  − 1.600 to − 0.179; *Z* =  − 1.566, *I*^2^ = 84.463, *p* > 0.05), and health profile (SMD = 0.041, 95% CI =  − 0.363 to 0.281; *Z* =  − 0.250759, *I*^2^ = 56.876%, *p* > 0.05). In subgroup analyses, it was determined that the types of psychotherapeutic interventions were not effective, but the number of sessions was effective.

**Conclusion:**

Psychotherapeutic interventions to reduce symptoms in endometriosis are effective in improving quality of life but not pain and health profile.

## Introduction

Endometriosis is a pathology that affects 5–15% of women of reproductive age [1], endometrial tissue grows in areas outside the uterine cavity and myometrium, forming nodules and masses, clinically manifested by symptoms such as pelvic pain and infertility [2]. The most common sites of endometriosis include ovaries, cecum, uterine broad ligament, uterosacral ligament, uterus, fallopian tubes, sigmoid colon, rectum, and appendix [3].

Endometriosis has negative consequences for both affected women and society, leading to an increase in hospitalizations, surgical interventions and costs related to infertility, pain, or anxiety treatment. It also reduces productivity and leads to labor force losses [4]. The disease negatively impacts young women’s social, career, academic, and economic potential and can lead to psychiatric problems such as severe, cyclical or chronic pain, anxiety disorders, and depression that last for many years [5, 6]. These symptoms reduce the overall quality of life of women, directly affecting the physiological, psychological, and social behavior of patients [2]. It takes an average of 8 years to diagnose endometriosis, and musculoskeletal disorders and psychological disorders may also develop due to endometriosis [7].

There is no definitive cure for endometriosis; therefore, the main aim of management strategies is to control the pain associated with the disease. This control is achieved through hormonal suppression of the disease or surgical excision. However, hormonal therapy can cause intolerable side effects and may become ineffective over time. The effect of surgical interventions is usually short-term [8]. Advances in the understanding of endometriosis have increased interest in less invasive and non-pharmacological treatment methods [8, 9].

In a meta-analysis to determine the impact of physical activity and exercise on endometriosis-related symptoms, the effects on pain, improvements in symptoms and quality of life could not be clearly determined due to limitations in the included studies [10]. However, in the meta-analysis conducted by Xu et al. (2017), the available literature suggests that acupuncture reduces pain, regardless of the control intervention applied. However, it is emphasized that the number of studies on this subject is quite limited [1]. Therefore, it is stated that additional blinded studies with appropriate control groups and adequate sample sizes should be conducted to confirm these findings. Therefore, the current study aims to fill an important gap in the literature by evaluating the effect of psychotherapeutic interventions on pain and quality of life in endometriosis.

## Methods

This planned study was conducted with the systematic review and meta-analysis method and was prepared under the guidance of the PRISMA checklist (Preferred Reporting Items for Systematic Reviews and Meta-Analyses Protocols) [11]. In order to increase the reliability of the study, the literature review, the selection process of the articles, and the data collection stages were carried out by three independent researchers. In addition, the quality assessment processes of the studies included in the study were meticulously examined and completed by the researchers. Thus, an effective approach was adopted to minimize the risk of potential bias in the research. This methodological rigor aims to increase the reliability of the findings.

### Inclusion and exclusion criteria

This systematic literature review (SLR) and meta-analysis includes data from randomized controlled trials (RCTs) examining the effect of psychotherapeutic interventions in women diagnosed with endometriosis. The studies focused on outcome measures such as pain and quality of life. All studies met the prespecified PICOS (population-intervention-comparators-outcomes-study design) inclusion criteria. The study aims to evaluate the potential effects of psychotherapeutic interventions on the mental and physical symptoms of women diagnosed with endometriosis.

Population (P): Women diagnosed with endometriosis.

Intervention (I): Psychotherapeutic intervention (yoga, exercise, manual therapy, hypnotherapy, mindfulness-based therapy, transcranial direct current stimulation, osteopathic manipulative therapy).

Comparison (C): No psychotherapeutic intervention or no intervention such as waiting list, control group.

Outcomes (O): Studies should report at least one of the following outcomes and measures of variation (e.g., SD, 95% confidence interval (CI)): change in endometriosis, health profile, quality of life, and pain levels.

Study design (S): Randomized controlled trials (RCTs) with relevant intervention or comparator, published in Turkish or English.

Exclusions: This study excluded letters to the editor, case reports, case reports, qualitative studies, descriptive studies, analytical studies, systematic and traditional reviews.

### Search strategy

Screening August–October 2024, MeSH eligible (“endometriosis”[MeSH Terms] OR “endometriosis”[All Fields]) AND ((“endometriosis treatment”[All Fields] OR “endometriosis management”[All Fields])) AND (“pain”[MeSH Terms] OR “pain”[All Fields])) keywords were used to search through EBSCOhost, Web of Science, PubMed, National Thesis Center of the Council of Higher Education, and Google Scholar, and the studies were transferred to Mendeley. No date limitation was imposed for the searches, and a search covering all years was performed.

### Selection of studies

**Fig. 1:**
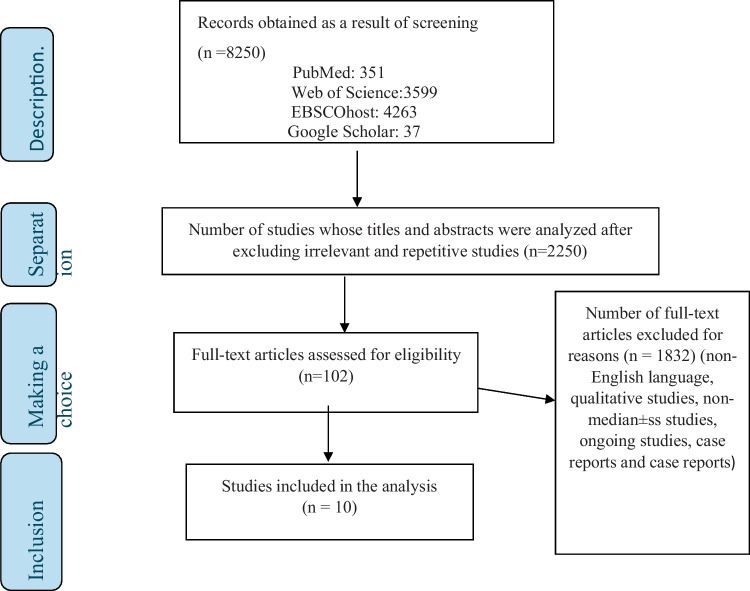
Selection of studies according to PRISMA flow diagram

As a result of the screening process, 8250 records were first accessed. At this stage, irrelevant and repetitive studies were sorted and eliminated. Then, the titles and abstracts of 2550 studies were subjected to a detailed review. As a result of this review, the full text of 102 studies was selected for review. The 102 articles were evaluated for compliance with the inclusion and exclusion criteria, and 10 studies investigating the effects of psychotherapeutic interventions on pain and quality of life in the treatment of endometriosis were analyzed. Detailed information about the selection process of the articles can be found in Fig. 1.

### Data extraction of study data

The researchers used a data extraction tool they developed to collect data on studies included in systematic reviews and meta-analyses. This tool enabled the studies to be classified in an orderly manner and included in the analysis process. The tool made it possible to systematically collect information such as mean and standard deviation values of the final test scores of the intervention and control groups in the studies, as well as data such as authors, year of publication, country of study, sample size, patient group, scales used, main findings, and quality score of the included studies (Table 1).

### Research ethics

This study was conducted by systematic review and meta-analysis method and was prepared in line with the relevant literature. Ethics committee permission was not required.

**Fig. 2:**
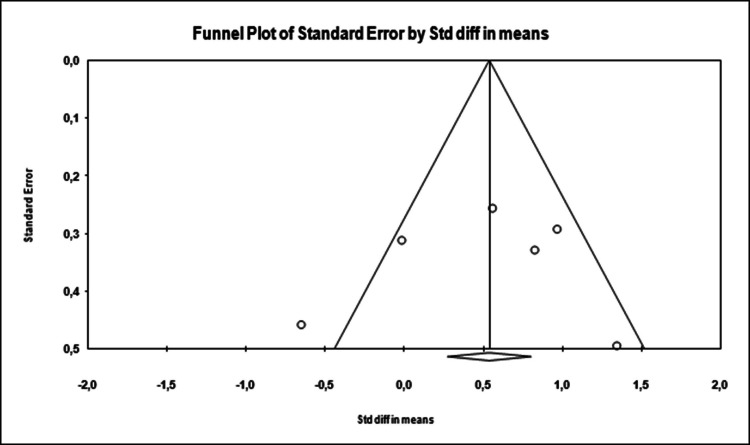
Funnel plot of studies reporting results on the effect of psychotherapeutic interventions on quality of life in endometriosis

### Assessment of methodological quality of studies

The quality assessment of the studies included in this systematic review and meta-analysis was performed with quality assessment tools according to the research design prepared by The Joanna Briggs Institute [12]. In our study, evaluation tools consisting of 13 questions for randomized controlled trials and 9 questions for quasi-experimental studies were used [12, 13]. The questions in these tools are answered with “Yes, No, Uncertain, Not Applicable” options. Methodological quality was assessed independently by two authors and consensus was achieved through a discussion. In this study, the evaluation results for each study are included in Table 1 as “Quality score.”

**Table 1:** Characteristics and results of the included studies

Author/Year	Type of intervention	Working pattern	Sample volumecharacteristic	Scale used	Main results	Interventiontime	Quality score
Moreira et al., 2024 [12]	Mindfulness-Based Intervention (bMBI)	Randomized controlled	Experimental group 31 Control group: 32	Short Form Health Survey (SF-36)	It was stated that psychosocial intervention could be added to the standard treatment plan.	4 sessions	Yes:8/13 No:4/13 Undetermined:1/1 3 Does not apply:0/13
Hansen et al., 2023 [13]	Specific Mindfulness- and Acceptance-Based Psychological intervention (MY- ENDO)	Randomized controlled	Experimental group: 19 Control group: 19	Endometriosis Health Profile (EHP-30)	Women with endometriosis may have significant and large effects of psychological intervention on QoL.	11 sessions	Yes:11/13 No:2/13 Uncertain:0/13 Does not apply:0/13
Mechsner et al., 2023 [14]	Transcranial Direct Current Stimulation	Randomized controlled	Experimental group: 18 Control group: 18	Numerical Rating Scale (NRS)	tDCS is an effective therapy for pain reduction	10 sessions	Yes:9/13 No:4/13 Uncertain:0/13 Does not apply:0/13
Muñoz- Gómez et al., 2023 [15]	Manual Therapy	Randomized controlled	Experimental group 21 Control group 20	Visual Analog Scales (VAS) Endometriosis Health Profile (EHP-30) Short Form Health Survey (SF-36)	Manual therapy can be a complementary treatment method in relieving pain and improving women's health profile and physical quality of life.	8 sessions	Yes:10/13 No:3/13 Uncertain:0/13 Does not apply:0/13
Shahriyaripoor et al., 2023 [16]	Hypnotherapy	Randomized controlled	Experimental group 10 Control group: 10	Visual Analog Scales (VAS)	Hypnotherapy in combination with medication is more effective than medication in relieving	8 sessions	Yes:11/13 No:2/13 Uncertain:0/13 Does not
					endometriosis pain.		apply:0/13
Hansen et al., 2017 [17]	Mindfulness-Based Psychological Intervention	Quasi- experimental	10 Patients	Endometriosis Health Profile (EHP-30) Short Form Health Survey (SF-36)	mindfulness-based psychological intervention is important for women with endometriosis and improves quality of life.	10 sessions	Yes:5/9 No:1/9 Uncertain:0/9 Not applicable:3/9
Gonçalves et al., 2016 [18]	Yoga	Randomized controlled	Experimental group:28 Control group:12	Endometriosis Health Profile (EHP-30)	Yoga practice was associated with a reduction in levels of chronic pelvic pain and an improvement in QoL in women with endometriosis.	16 sessions	Yes:5/9 No:4/9 Uncertain:0/9 Does not apply:0/9
Darai et al., 2015 [19]	Osteopathic Manipulative Therapy (OMT)	Quasi- experimental	20 Patients	Short Form Health Survey (SF-36)	OMT improves quality of life.	24 sessions	Yes:5/9 No:4/9 Uncertain:0/9 Does not apply:0/9
Petrelluzzi et al., 2012 [20]	Physical therapy and psychological intervention	Quasi- experimental	26 Patient	Visual Analog Scales (VAS) Short Form Health Survey (SF-36)	The physical and psychological intervention was effective in reducing perceived stress, normalizing cortisol levels, increasing vitality and improving physical functioning.	10 sessions	Yes:5/9 No:1/9 Uncertain:0/9 Not applicable:3/9
Kold et al., 2012 [21]	Mindfulness-based psychological intervention	Quasi- experimental	10 Patients	Endometriosis Health Profile EHP-30 Short Form Health Survey (SF-36).	The study is important in terms of raising women's awareness. It is recommended that more studies be conducted.	10 sessions	Yes:5/9 No:4/9 Uncertain:0/9 Does not apply:0/9

### Data synthesis

For the statistical calculations of the study, CMA Ver. 2. was used. Heterogeneity between the studies was assessed using the chi-squared statistic and Higgins *I*^2^ tests, and an *I*^2^ greater than 50% was considered to indicate significant heterogeneity. Studies with *I*^2^ > 50% and *p* value >.1 were evaluated using the random effects model, while studies with *I*^2^ ≤ 50% and *p* value >.1 were evaluated using the fixed effects model [14].

The tau-squared statistic was used to complete the assessment of variance and heterogeneity between studies. The standardized mean difference (SMD) with 95% CI was also used to assess the effect size associated with the same outcome with different measurement tools. Forest plots were also prepared to visualize the SMD with 95% CI. These *D* values were averaged to calculate the overall effect size. The *D* value was converted to a *Z* value to assess its statistical significance. Funnel plots were used to examine and visualize publication bias. Egger’s test was also performed to assess publication bias. All values were two-sided and were considered significant at a threshold of 0.05 [15].

The chi-square test and Higgins *I*^2^ statistic were used to assess heterogeneity across studies. These tests give an idea of how generalizable the results are, along with assessing the consistency across studies. An *I*^2^ value above 50% indicates that the results of the meta-analysis may vary due to different populations or study conditions, and therefore, a random effects model may be more appropriate [14].

The tau-squared statistic was performed to further assess the degree of variance and heterogeneity between studies. This statistic helps us to determine how much of the heterogeneity is due to random variation. SMD was done to compare the effects of the measurement tools used in different studies. Forest plots were created to visualize these SMD values.

## Results

The titles and abstracts of the scanned articles were first screened. Mendeley was used to remove duplicates and organize the reference list.

Six of the studies included in the study were randomized controlled experimental and three were quasi-experimental. The total sample size of the studies was 304 (intervention group = 127; control group: 111; single group = 66) (Table 1).

All of the studies included in this systematic review and meta-analysis were found to meet more than 50% of the quality of evidence assessment tool items (Table 1). This result is important in terms of showing that the information presented in our study is based on studies with an acceptable level of evidence quality.

### Meta-analysis results on the effectiveness of psychotherapeutic interventions on quality of life in endometriosis

In this study, two methods were used to determine whether there was publication bias.

1. Funnel scatter plot
2. Tested with Egger’s regression test [16]

In the funnel plot, which is one of the important methods indicating publication bias, it was determined that the studies showed a symmetrical distribution in the middle of the funnel. This shows that there is no publication bias (Figure 2).

Egger’s method was used as the second method to determine whether there was publication bias. According to Egger’s method, the cutoff point (B0) was 3.62848, 95% confidence interval (− 11.09600 to 9.05253), *t* = 0.28159, *df* = 4, and two-way *p* value was 0.79223. This result shows that there is no statistical publication bias (*p* = 0.45228).

**Fig. 3:**
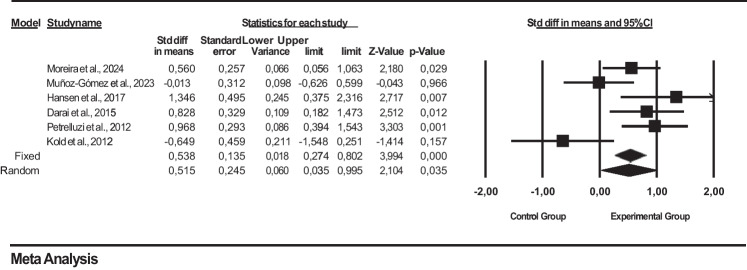
Forest plot for the effect of psychotherapeutic interventions on quality of life in endometriosis

Figure 3 shows the effect sizes, standard error, variance, lower and upper limits, and forest plot of 6 studies on the effect of psychotherapeutic interventions used in endometriosis patients on quality of life. Short-Form Health Survey (SF-36) was used to assess quality of life in the studies. In a meta-analysis based on the findings of these studies, psychotherapeutic interventions were found to be effective on the quality of life of women with endometriosis (SMD = 0.515, 95% CI = 0.035 to 0.995; *Z* = 2.104, *p* = 0.035, Figure 3). The collected data showed that psychotherapeutic interventions had an overall significant effect on the quality of life of women with endometriosis in favor of the intervention group, with a moderate heterogeneity between studies (*I*^2^ = 67.523%; *p* = 0.009). Random was used in the heterogeneity test because *p* < 0.05.

### Meta-analysis results on the effectiveness of psychotherapeutic interventions on pain in endometriosis

**Fig. 4:**
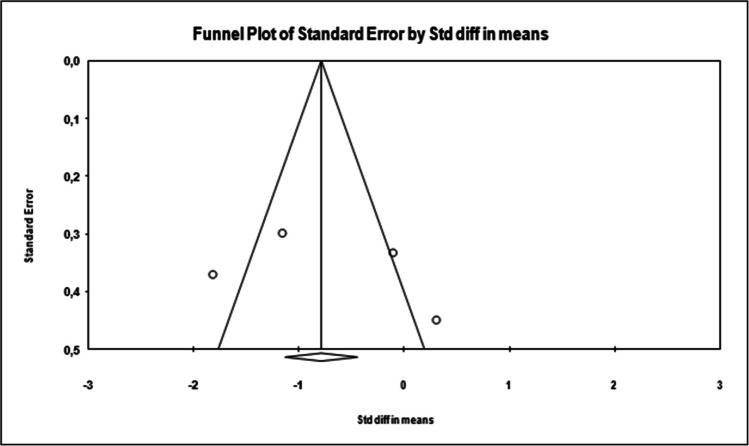
Funnel plot of studies reporting results on the effect of psychotherapeutic interventions on pain in endometriosis

In the funnel plot, which is one of the important methods indicating publication bias, it was determined that the studies were symmetrically distributed in the middle of the funnel. This shows that there is no publication bias (Figure 4).

Publication bias among the studies in this dataset was determined by Egger’s method. According to Egger’s method, the cutoff point (B0) is 5.86014, 95% confidence interval (− 36.91358 to 48.63387), *t* = 0.58948, *df* = 2, and two-way *p* value is 0.58948. This result shows that publication bias is not statistically significant (*p* = 0.58948).

**Fig. 5:**
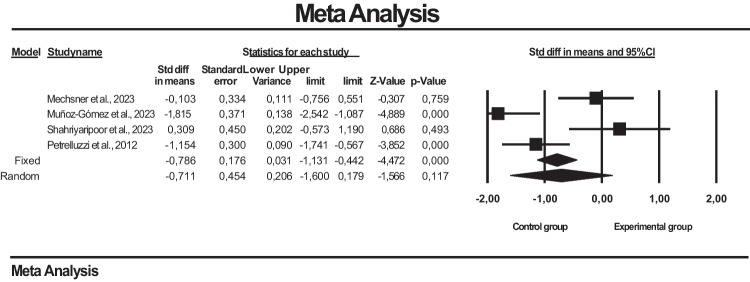
Forest plot for the effect of psychotherapeutic interventions on pain in endometriosis

Figure 5 shows the effect sizes, standard error, variance, lower and upper limits and forest plot of 4 studies on the effect of psychotherapeutic interventions on pain in endometriosis. Visual Analog Scale (VAS) and Numerical Rating Scale (NRS) were used to assess pain in the studies. In the meta-analysis based on the findings of these studies, it was found that psychotherapeutic interventions applied in endometriosis had no effect on pain and a high heterogeneity was detected between the studies (SMD = − 0.454, 95% CI = − 1.600 to − 0.179; *Z* = − 1.566, *p* = 0.117, *I*^2^ = 84.463) (Figure 5). Random was used in the heterogeneity test because *p* < 0.05.

### Results of a meta-analysis on the effect of psychotherapeutic interventions on the health profile in endometriosis

**Fig. 6:**
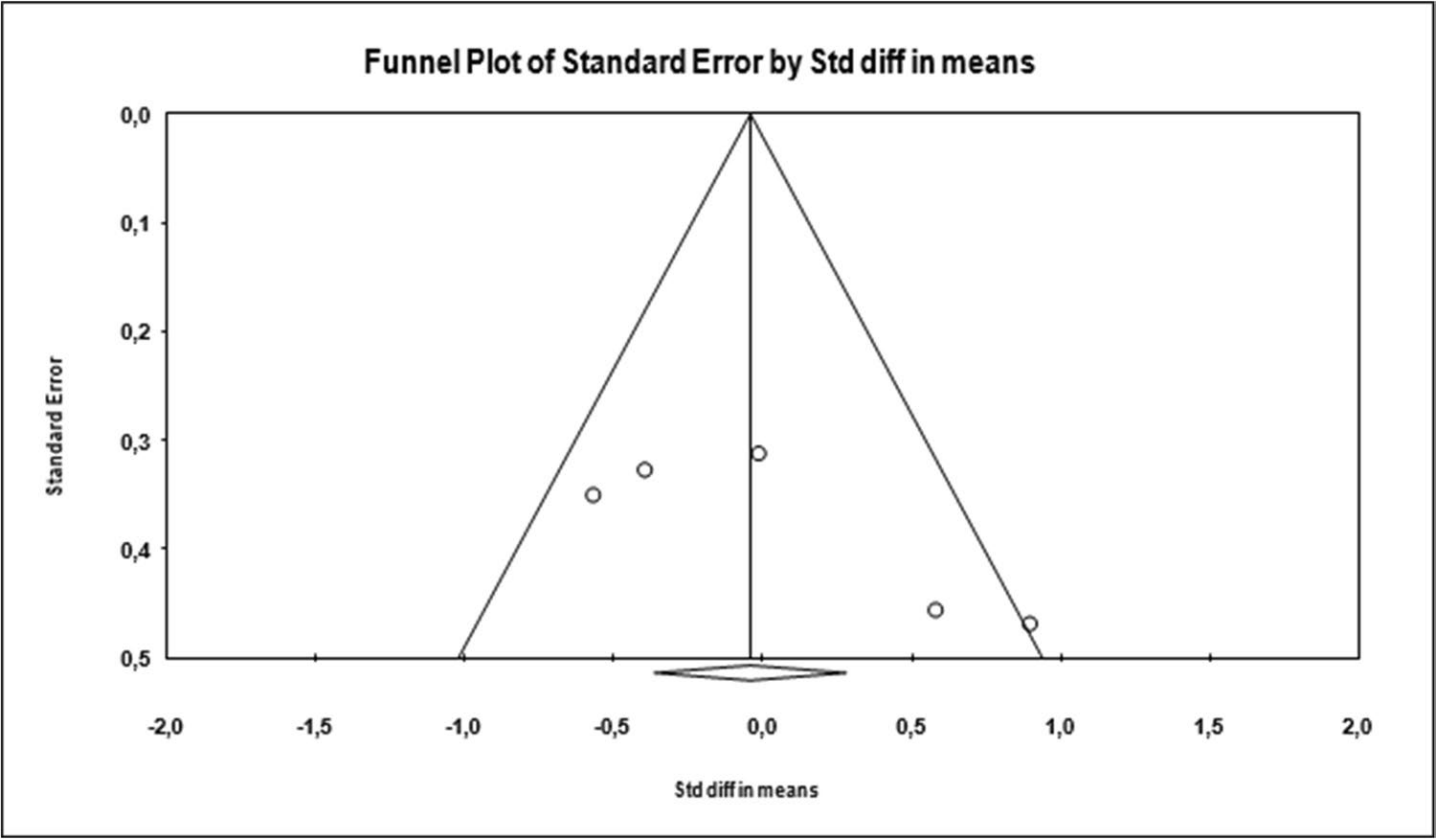
Funnel plot of studies reporting results on the effect of psychotherapeutic interventions on health profile in endometriosis

In the funnel plot, which is one of the important methods indicating publication bias, it was determined that the studies in this data set showed a symmetrical distribution in the middle of the funnel. This shows us that there is no publication bias (Figure 6).

Publication bias among the studies in this dataset was determined by Egger’s method. According to Egger’s method, the cutoff point (B0) = 6.69161, 95% confidence interval (− 2.55424 to 15.93746), *t* = 2.30327, *df* = 3, and two-way *p* value is 0.10467. This result shows that publication bias is not statistically significant (*I*^2^ =56.876; *p* = 0.055).

**Fig. 7:**
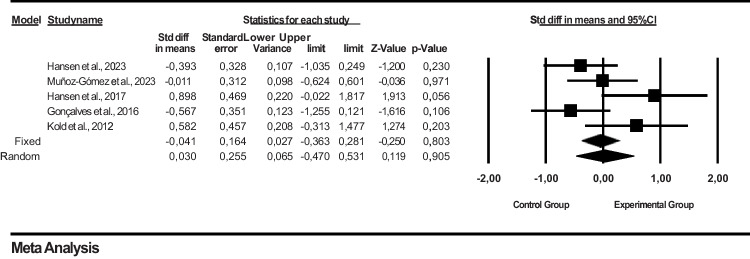
Forest plot for the effect of psychotherapeutic interventions on health profile in endometriosis

Figure 7 shows the effect sizes, standard error, variance, lower and upper limits, and forest plot of 5 studies on the effect of psychotherapeutic interventions on health profile in endometriosis. Endometriosis Health Profile (EHP-30) scale was used to evaluate the effect on health profile in the studies. In a meta-analysis based on the findings of these studies, it was found that psychotherapeutic interventions had no effect on the health profile (SMD = − 0.041, 95% CI = − 0.363 to 0.281; *Z* = − 0.250, *p* = 0.803) (Figure 7). The collected data showed that mindfulness-based cognitive therapies had an overall significant effect on depression level in women in favor of the intervention group, and a fixed effects model was applied for the studies included in the meta-analysis (*I*^2^ = 56.876%; *p* = 0.055). Fixed was used in the heterogeneity test because *p* > 0.05.

The average effect size value for the number of interventions was 0.303 and *p* < 0.05. In the study, it was determined that the psychotherapeutic interventions played a role on the number of sessions. Twenty-four and 4 sessions were found to be effective in practice. In addition, the average effect size of the type of psychotherapeutic intervention applied in the meta-analysis was calculated as 0.061 and *p* >.05. It was determined that the type of psychotherapeutic intervention did not change the effect size of psychotherapeutic interventions applied to individuals in general. However, hypnotherapy, osteopathic manipulative therapy, physical therapy, and psychological interventions showed an effect (Table 2).

**Table 2:** Moderator results for the effect of psychotherapeutic interventions on quality of life in endometriosis

Moderator	Number of studies	Impact size	Standard error	Lower limit	Upper limit	*p*
**Number of sessions**						
4 sessions	1	0.560	0.257	0.056	1.063	**0.029**
8 sessions	2	-0.819	0.807	-2.400	0.763	0.310
10 sessions	4	0.390	0.427	-0.446	1.226	0.361
11 sessions	1	-0.393	0.328	-1.035	0.249	0.230
16 sessions	1	0.102	0.448	-0.775	0.979	0.820
24 sessions	1	0.828	0.329	0.182	1.473	**0.012**
**Total**	12	0.303	0.148	0.013	0.593	**0.041**
**Type of intervention**						
Hypnotherapy	1	-1.627	0.320	-2.254	-1.00	**0.001**
Mindfulness	4	0.200	0.399	-0.581	0.981	0.616
Manual Therapy	1	-0.013	0.312	-0.626	0.599	0.966
Osteopathic Manipulative Therapy (OMT)	1	0.828	0.329	0.182	1.473	**0.012**
Physical therapy and psychological intervention	1	0.968	0.293	0.394	1.543	**0.001**
Transcranial Direct Current Stimulation	1	-0.103	0.334	-0.756	0.551	0.759
Yoga	1	0.102	0.448	-0.775	0.979	0.820
**Total**	10	0.061	0.128	-0.190	0.311	0.635

## Discussion

### Quality of life

In this study, evaluating the effectiveness of psychotherapeutic interventions on endometriosis, improvement in quality of life was observed in women. The studies included in this systematic review and meta-analysis used the SF-36 to assess quality of life. A previous systematic review also showed similar results to our study, showing improvements in the physical function and vitality items of quality-of-life questionnaires [17]. In the meta-analysis by Abril-Coello et al. (2022), in which they evaluated the efficacy of physical therapy including 6 studies, significant results were obtained only for the physical function sub-variable related to quality of life (SMD − 1.49; 95% CI between − 2.88 and − 0.10; *I*^2^ = 95%). No statistically significant differences were found for the other variables analyzed [18]. Quality of life is a variable represented by many factors such as pain severity, social aspects, emotional roles and physical function. Study results suggest that conservative therapies may have a greater impact on the physical function variable. These results suggest that psychotherapeutic interventions such as physical exercise, yoga, acupuncture, manual therapy improve quality of life in women with endometriosis.

Furthermore, in this meta-analysis, the types of psychotherapeutic interventions applied were found to change the effect size and were statistically significant. In particular, OMT and Physical therapy and psychological intervention were statistically significant compared to manual therapy, hypnotherapy, mindfulness-based therapy, transcranial direct current stimulation, and yoga.

### Pain

In this study, evaluating the effectiveness of psychotherapeutic interventions on endometriosis, we found a decrease in pain levels only in 2 studies in which physical therapy and psychological intervention and manual therapy were applied as interventions [19, 20]. In other studies, no change in pain level was observed. VAS and NRS were used to assess pain in the studies included in this systematic review and meta-analysis. In the meta-analysis of 10 studies by Xu et al. (2017), the mean difference (MD) in pain reduction (difference in pain levels before and after intervention, measured on a 0–10 point scale) between the acupuncture and control groups was 1.36 (95% CI = 1.01 ± 1.72, *p* < 0.0001) [1]. In another meta-analysis including six studies evaluating physical therapy, significant results were obtained for pain severity (SMD − 0.89; 95% CI − 1.21 to − 0.57; *I*^2^ = 69%) [21]. In the meta-analysis of Le Liu (2020), which included 11 studies involving 776 patients diagnosed with endometriosis, the results show that acupuncture is effective in relieving pain associated with endometriosis [22]. In the meta-analysis by Tennfjord et al. (2021), flexibility and strength training, cardiovascular fitness and yoga were found to improve pain severity in only one study. However, due to the heterogeneity of study results and measurements and confounding factors, a quantitative meta-analysis could not be performed [23].

### Health profile

In this study, analyses evaluating the effectiveness of psychotherapeutic interventions on endometriosis showed no significant change in women’s health profiles. The studies included in this systematic review and meta-analysis used the Endometriosis Health Profile (EHP-30) to assess the health profile. As the first study to evaluate the health profile, it is an important starting point. These results may be due to the limited effects of psychotherapeutic interventions on physical symptoms or the lack of a physical treatment-oriented approach with psychological support. In addition, it should be taken into consideration that women’s health status varies individually and therefore it may be misleading to expect a generalized effect.

## Conclusion

The results of this systematic review and meta-analysis show that psychotherapeutic interventions for the treatment and management of endometriosis are beneficial in terms of quality of life. In addition, the study found that psychotherapeutic interventions for the treatment and management of endometriosis were not effective on pain and health profile.

In the study, it was found that the types of psychotherapeutic interventions were not effective, but the number of sessions (4 sessions and 24 sessions) was effective in practice.

It should be kept in mind that it may be difficult to reach definitive conclusions in the evaluation of the effectiveness of psychotherapeutic interventions in patients with endometriosis due to significant differences between studies in terms of the scales used in the studies and their sensitivity and specificity, time for measurements and follow-up period. Further randomized controlled trials are needed to determine whether psychotherapeutic interventions for the treatment and management of endometriosis are beneficial in terms of pain and health profile.

### Limitations

It is thought that the small sample size of some of the analyzed studies may reduce the strength of evidence of the results of the study.

## Data Availability

The datasets used and/or analyzed during the current study are available from the corresponding author on reasonable request.
